# Case report: Intensive online trauma treatment combining prolonged exposure and EMDR 2.0 in a patient with severe and chronic PTSD

**DOI:** 10.3389/fpsyt.2024.1370358

**Published:** 2024-04-22

**Authors:** Suzy J. M. A. Matthijssen, Sophie D. F. Menses

**Affiliations:** Altrecht Academic Anxiety Centre, Altrecht GGZ, Utrecht, Netherlands

**Keywords:** PTSD, EMDR 2.0, prolonged exposure, online, case report

## Abstract

**Introduction:**

Short and intensive trauma treatment programs seem promising in treating post-traumatic stress disorder (PTSD). However, little is known about the effects performing these types of intensive treatment programs online.

**Method:**

At the Altrecht Academic Anxiety Centre, an in person intensive trauma focused treatment of six days (three consecutive days in two weeks) was altered into a fully online treatment. A treatment day consisted of 90 minutes of prolonged exposure, 60 minutes of exercise, 90 minutes of Eye Movement Desensitization and Reprocessing (EMDR) 2.0 and 60 minutes of psychoeducation. Mary, a patient diagnosed with chronic and severe PTSD, chronic depressive disorder (single episode, moderate to severe), a panic disorder, and an other specified personality disorder was the first patient to take part in this intensive online trauma treatment.

**Results:**

Mary reached full remission of PTSD. The PTSD symptoms (measured on both the clinician-administered PTSD scale for DSM-5, CAPS-5 and The PTSD Checklist for DSM-5, PCL-5) showed maximum improvement and were completely absent during one month and six month follow-up. Moreover, she no longer suffered from severe depressive symptoms and did not report any general psychiatric symptoms (measured with the Beck Depression Inventory version 2, BDI-II and the Brief Symptom Inventory, BSI).

**Conclusion:**

In conclusion, the case-report demonstrates that intensive trauma treatment online was successful in this specific case, thereby being a ‘proof of concept’ that intensive trauma treatment online is feasible. It might be promising for patients with severe and chronic PTSD and comorbid psychiatric disorders. However, further research must show if the results of this specific case can be translated to other patients with severe and chronic PTSD and comorbid psychiatric disorders.

## 1 Introduction

Post-traumatic stress disorder (PTSD) is a debilitating disorder, with a lifetime risk of developing estimated at 6.8%, and women more likely to be affected than men (1). In high-risk populations the prevalence of PTSD is even estimated at 15.4% (2). The disorder is manifested through symptoms of re-experiencing, hyper-arousal, negative cognitions and feelings, and avoidance following a traumatic event (Diagnostic Statistical Manual- 5; DSM-5) (3). Fortunately, there are a number of effective PTSD treatments among which are Prolonged Exposure (PE) and Eye Movement Desensitization and Reprocessing (EMDR) (4), but nonetheless, PTSD treatments still show fair dropout and non-response rates which emphasizes the need for optimization of therapies [e.g. (5)]. In an effort to reduce PTSD symptoms in a substantially shorter time, brief and highly intensive treatment programs have been developed [e.g (6–9)]. Treatment results of these programs are promising, as similar treatment outcomes have been found when compared to regular trauma-focused therapies (10), while there are no reports of symptom exacerbation or increased dropout rates. Smaller studies of Ehlers et al. (7) and Hendriks et al. (8) report 0% dropout, Wagemans et al. (11) report a dropout rate of less than 4% and Matthijssen et al. (9) of 8.2%, which is less than the reported average of 16 to 18% (5, 12). So, short and intensive treatment programs could be a valuable addition to the existing range of regular spaced trauma-focused therapies.

Treatment of PTSD generally takes place in an in person setting. Although online treatment methods did exist, it was not until the coronavirus disease (COVID)-19 pandemic, which led to restricted possibilities for face-to-face psychological interventions due to imposed social distancing and (partial) lockdowns, that the use of online interventions took off. Morland et al. (13) found that evidence-based PTSD treatments delivered through office-based and home-based clinical video conferencing consistently demonstrated feasibility and acceptability as well as significant reductions in PTSD symptoms, non-inferior outcomes, and furthermore, also when compared with traditional face-to-face office-based care, comparable dropout rates. Also, other issues can be overcome with telehealth interventions such as travel time and costs, privacy concerns and physical difficulties.

**Figure 1:**
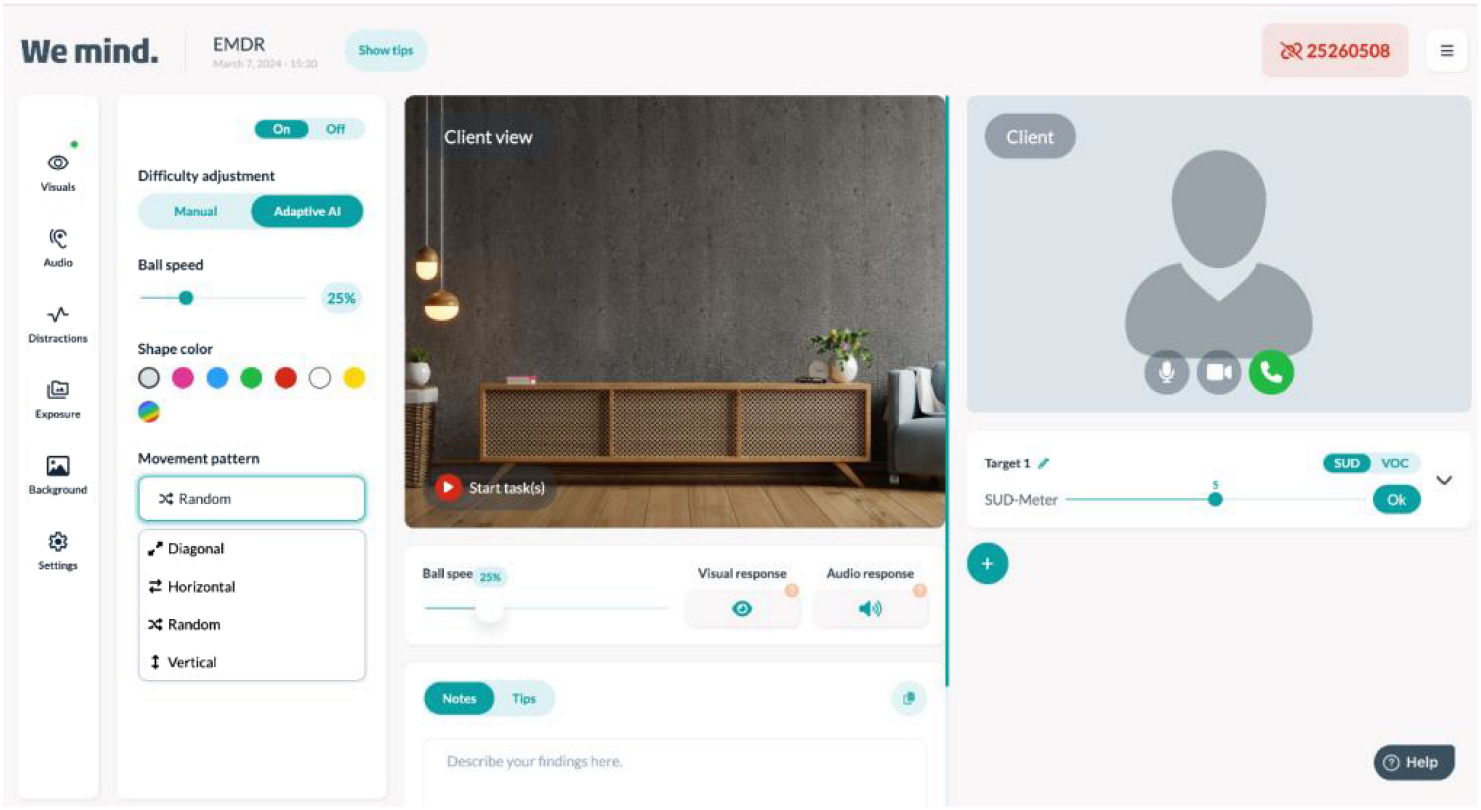
Visual Display of the therapist view of the EMDR online tool. Reproduced with permission.

Until recently, combining intensive trauma treatment and telehealth had never been done. In the early beginning of the COVID pandemic, at the Altrecht Academic Anxiety Centre, an in person intensive trauma focused treatment of six days (three consecutive days in two weeks) was altered into a fully online treatment. The daily program consisted of 90 minutes PE, 60 minutes of physical exercise, lunchbreak, 90 minutes of EMDR (version EMDR 2.0) (14) and 60 minutes of psychoeducation. Also, homework exercises were given to the patient to practice with triggers and/or to break through avoidance. PE and EMDR are two evidence based forms of trauma focused treatment (15, 16). PE is a trauma treatment where the patient tells the traumatic event over and over again in order to falsify the harm expectancy of what could happen if the patient indulges him or herself in the traumatic memory (9). EMDR is a form of trauma treatment where the patient is asked to keep the most disturbing image of the traumatic memory in mind and dual working memory taxation is offered to compete with holding the disturbing image with the same emotional disturbance. The memory loses disturbance and is stored in this altered way (17) EMDR 2.0 is an enhanced form of EMDR which has three main elements; motivation, activation and desensitization. Motivation is focused on giving the patient proper information about EMDR and the supposed working mechanism (dual taxation) and explain that the patient has a role in keeping the memory activated during treatment. Activation is focused on helping and instructing the patient to keep the memory activated and lastly, desensitization, which is focused on optimizing dual taxation and also implementing modality specific dual taxation if necessary (14, 18) behavior. Every treatment day one traumatic memory was targeted. PE was delivered through video conferencing, EMDR 2.0 was delivered with an online EMDR tool also including video conferencing (19). In the EMDR tool, the participant had to follow a moving digital ball and respond to the ball changing into a cylinder by pressing a specific button on the keyboard. The therapist could change the amount of dual taxation (e.g. changing the speed of the ball or the speed of changing to cylinder, make the ball changing in color, add auditory taxation) (19). For a visual display see **Figure 1**. Physical exercise was conducted by the patient at home in front of the television while watching a selected YouTube fitness video. Psychoeducation was prerecorded and patients were phoned after watching the prerecorded psychoeducation to inform if there were any remaining questions. In the current case report the results of the first patient conducting the intensive online trauma treatment program are described. More information on the regular intensive program and the trauma therapies can be found in Matthijssen et al. (9).

To the best of our knowledge there are only two studies reporting about the effects of an online intensive trauma focused treatment in which PE and EMDR(2.0) are combined. In one case report (20) six patients suffering from PTSD (of which four with Complex PTSD; CPTSD) received four consecutive days of treatment and the results showed four of the six patient lost their PTSD or Complex PTSD diagnosis and scores on a PTSD interview (CAPS-5) and self-reported PTSD symptoms (PCL-5) decreased significantly. Research from the same treatment facility recently also showed in a sample of 73 patients that clinical (CAPS-5), self-reported (PCL-5), and Complex PTSD symptoms (International Trauma Questionnaire – Disturbances in Self Organization; ITQ-DSO) decreased and that 82,2% of the patients no longer met PTSD criteria (21). Interestingly also, in both studies no dropout and no adverse events occurred.

The aim of this case report is to increase knowledge about the feasibility and effectiveness of an intensive online trauma focused treatment program, with a combination of EMDR 2.0 and PE, but also to provide insight in how such a treatment is done specifically. In the current case study we describe a treatment of a woman with severe and chronic PTSD due to severe childhood trauma. Symptoms of PTSD, depression and general psychiatric symptoms were measured at screening, pretreatment, posttreatment, after one week, one month and six months follow-up using both self-report measures and for PTSD also a clinical measure.

## 2 Case description

Mary, a 45 year old women, was born in Mexico. Her biological mother was not able to take care of her and her two brothers (+2, -4). She was brought to a niece of her mother who was very poor, and who treated her and her brothers badly and often outsourced the parental duties to a deaf-mute cousin. Mary often wandered the streets looking for food. She stayed in a children’s home for a while and from there she was placed with a foster family. In the children’s home, the boys got fed, but the girls got less food or sometimes even no food. At the age of 5 Mary was, together with her brothers, adopted by a Dutch family. Upon arrival in the Netherlands, she was severely malnourished and apathetic. She didn’t speak well and wasn’t used to playing. She was stimulated very little and had difficulty keeping up with school. There was a lot of fighting and violence in the adoptive family and on top of that, Mary was sexually abused both by one of her biological brothers as well as by her adoptive father. She attended a school for children with learning disabilities and she learned to read and write there. Until today, she still has difficulty reading, writing and calculating. When she finished high school she started working full-time at a burger restaurant, and after that she worked as a housekeeper at a nursing home. Here she was asked to work as a caretaker for elderly patients and this is now her current job. The job is quite stressful due to the shortages of care workers. She is involved in a relationship with a man by whom she feels supported. They are living together. Previous relationships were not a positive contributor for her feelings of self-worth, trust and image of herself.

Mary’s first contact with mental health care was at the age of 16. The reason for this encounter was the fear of going home, because of the daily abuse by her adoptive father and brother. However, she never reported the abuse by her brother. She had several treatment sessions over the course of one year. The type of therapy is unknown. The sessions did not have any effect. At the age of 19 Mary had creative therapy, at 29 she completed an assertiveness training. From age 30 to 32 she had weekly supportive sessions with a psychotherapist. All therapies were unsuccessful in alleviating trauma symptoms. From age 35 until 37 she followed cognitive behavioral therapy for anxiety symptoms, partially in group sessions, but mostly individual. This therapy was not aimed at treating trauma symptoms. The therapy was stopped after a switch to another therapist, with whom she had less connection. Mary was referred to our clinic for the treatment of severe and chronic PTSD. Mary suffered from all PTSD symptoms except flashbacks, irritable behavior/anger outbursts and reckless or self-destructive behaviors. She had several forms of avoidance behaviors (e.g. she was unable to see herself naked in the mirror, go out on the street at night by herself or say words that are sexual or intimate). She also fulfills the criteria of a chronic depressive disorder (single episode, moderate to severe), a panic disorder and an other specified personality disorder (with borderline, dependent and avoidant traits). She used Citalopram, 25 mg. and Oxazepam, 10 mg., the last one irregularly when she deemed it necessary. She did not use any alcohol or drugs. Her goals for therapy were that she wanted to be less overwhelmed by memories of her past, and that she wanted to have fewer nightmares about her father. She also said “I want to live my life without it being dominated by fear”. Mary had a long list of traumatic events (not all meeting A-criteria PTSD); see **Table 1**. Assessment and procedure.

**Table 1:** History of life-events of Mary.

Age	Event(s)
*0-5*	*Neglect by birth parents (Mexico)*
*5*	*Adoption*
*From 11-12*	*Sexual abuse by adoptive father*
*From 14*	*Sexual abuse by half-brother*
*5-15*	*Beaten by adoptive mother and treated as a slave*
*16-19*	*Beaten by adoptive father*
*18*	*Witnessing an armed man at work*
*20*	*Forced to perform sexual acts with her ex-partner*
*20*	*Sudden death of 22 year old brother (went missing and was found dead)*
*23*	*Witnessing an armed man at work for a second time*
*30*	*Being robbed and assaulted*
*37*	*Experiencing a fire*
*37*	*Being drugged and raped and as a consequence needed to undertake an abortion*

### 2.1 Clinical measures

The Dutch version (22) of the Life Events Checklist for DSM 5 (LEC-5) (23) was administered to assess adverse life events that meet the A-criterion of PTSD. A semi-structured clinical PTSD interview was used to determine the presence and severity of PTSD symptoms over the past week and/or month [CAPS-5 (24); Dutch version (25)]. Psychometric evaluation of the Dutch CAPS-5 showed adequate reliability and validity (26). The CAPS-5 month version was administered at screening, one month and six months after treatment. One week after treatment, symptoms were assessed over the past week. A self-report questionnaire for PTSD [PCL-5 (27); Dutch version (28)] was also used to assess the severity of PTSD symptoms over the past week. Psychometric assessment shows strong validity and reliability (29). Additionally, Mary was asked to what extent she suffered from her PTSD symptoms in daily life. She indicated her answer on a Visual Analogue Scale [VAS (30)] ranging from 0 (not at all) to 100 (extremely bothered). Depressive symptoms over the past week were measured using the Beck Depression Inventory [BDI-II-NL (31, 32)]. Another questionnaire was administered to measure general psychiatric symptoms over the past week [BSI (33, 34)]. All questionnaires were administered at screening, pre-treatment (at the beginning of the first day of treatment), posttreatment (at the end of the last day of treatment) and at follow-up after one week, one month and six months.

### 2.2 Procedure

The intensive trauma treatment program was provided online by the Altrecht Academic Anxiety Centre, a center specialized in the treatment of severe anxiety disorders, OCD and trauma related disorders, in Utrecht, the Netherlands. Before she entered treatment, Mary was screened on diagnoses and exclusion criteria for participating in the program. Inclusion criteria for the intensive treatment are an established PTSD diagnosis (according to the DSM-5), and having experienced multiple trauma’s (at least four A-criterion trauma events). Sedative medication needs to be reduced to a minimum, alcohol and drugs-use are prohibited during treatment up until one month follow-up. Exclusion criteria are a severe acute suicide risk, non-proficiency of the Dutch language, and/or severe psychiatric symptoms that could interfere with trauma treatment. In the case of Mary, the use of an SSRI (Citalopram, 25 mg.) was continued, but sedative medication (Oxazepam, 10 mg.), which she would take irregularly in low dosages, was discontinued. Also, a treatment plan was made. She was asked which 6 traumatic memories were most distressing at this moment. These memories would be the memories targeted in PE and EMDR 2.0 sessions during treatment [one memory each treatment day, ordered by Subjective Units of Disturbance (SUD) from high to low (35)]. Mary originally would have started PTSD treatment in the in person setting. However, due to COVID regulations implied by the national government the program was not proceeded in an in person setting and all treatments were switched as much as possible to remote treatment. This remote treatment was offered to Mary. She could also opt to wait for in person treatment until regulations were lifted (not knowing up front of course how long that would be), but she preferred to start the remote treatment. She was informed that the treatment was off label, and that there was limited evidence available about the effect of remote trauma treatment, let alone intensive remote trauma treatment. For that reason, she would be monitored closely, and she would serve as a pilot. Instructions on how to watch the psychoeducation and the exercise videos, how to use the online video program and the online EMDR tool were given. Treatment consisted of 3 hours of individual trauma focused treatment (90 minutes PE and 90 minutes EMDR 2.0 therapy), 60 minutes of physical activity and 60 minutes of psychoeducation per day (17). Sessions were provided by therapists who were trained in PE and EMDR 2.0 therapy.

### 2.3 Treatment outcomes

See **Table 2** for the results on all outcome measures. Mary selected six memories to work on during the intensive therapy. All memories selected caused a nine or ten out of ten on a subjective units of disturbance (SUD) scale, which ranges from zero (no disturbance at all) to ten (maximum disturbance) and were treated in order from highest to lowest SUD. The first day of treatment she started with the memory of sexual abuse by her adoptive father. Mary was nervous to start, but at the same time relieved and happy that the treatment could start. In the first PE session the formulated harm expectancy was that the anxiety that would come up during the session would never go away anymore. Mary sometimes dissociated but when she was called by her name it helped her to stay in the present moment. During the session it appeared the memory actually consisted of two events and a start was made with the one that gave the most distress. Even though the connection got interrupted in the middle of the session Mary was able to log in again and she could pick up where the session was interrupted. The harm expectancy was violated because at the end of the session she noticed the anxiety decreased. In the EMDR session three different targets were desensitized, all representing different sexual acts. Also cognitive interweaves (an intervention in which the therapist asks a question to the patient that is expected to elicit an answer that contributes to initiating stagnated information processing) were performed to target the feeling of guilt that was experienced by Mary. As homework exercise she had to watch a picture of her father three times for a duration of 30 seconds. The second day she worked on a memory of sexual abuse by her brother. She experienced in the PE that by repetition the memory felt more distant. Although the distress was maintained at quite a high level, she was able to cope better with the distressing memory. During the EMDR 2.0 therapy, not only the second memory was desensitized, but also the third memory, about her mother beating her. Homework for the day was looking at pictures of her brother, looking at her own body, and wearing a dress. She believed wearing one might provoke a new rape. At day three, Mary expressed the treatment was heavy, but she also noticed amelioration of symptoms. Homework exercises were partially done. The third memory was checked and although Mary was tense to get started, there was no distress anymore upon recalling the memory during PE. A start was made on the fourth memory; one where she is wandering the streets bear feet at age 3-4, looking for food, because there was nothing to eat. The distress during the PE session stayed high. In the EMDR 2.0 session the distress was decreased to a SUD score of zero. Homework exercises, apart from repeating earlier homework, also consisted of looking at pictures of herself at a very young age. During the weekend Mary practiced with her homework and her harm expectancies (being raped and not being able to cope with the triggers) were violated. The fourth day she worked on a memory in which she was dragged out of bed and locked up in her room by her mother, while she needed to use the toilet. Distress decreased during the PE and Mary felt relief at the end of the session, and she realized it was not her fault. During EMDR 2.0 the most prominent target was fully desensitized with the use of cognitive interweaves and rescripting, despite loss of the internet connection a few times. On day five of the treatment Mary reported back that she had practiced with her homework in which she had looked at herself in the mirror and was able to wash herself while showering. The target memory on the fifth day was about physical abuse by her father. During PE the distress remained quite high, about which Mary concluded that she was able to cope with it. Although there was a short loss of internet connection again, three targets were desensitized during EMDR 2.0. Homework exercises were looking at old pictures of her father and going into the supermarket while remaining close to people that look like her father (instead of avoiding them). Harm expectancies (about not being able to cope with the memories that would come up) were violated. On the last day of treatment a memory was treated in which Mary made a mistake and was yelled at by her father, after which she went crying in her room. Distress during PE decreased. During EMDR 2.0 two targets within this memory were desensitized, and an additional last memory was also desensitized. Lastly, a mental video check (walking through a future situation imaginally and desensitizing triggers) was done on a situation in which Mary would be asked to perform a difficult task.

**Table 2:** Raw scores.

	Screening	Pre-treatment	Post-treatment	One week follow-up	One month follow-up	Six month follow-up
CAPS-5 (month)	45	n.a.	n.a.	n.a.	0	0
CAPS-5 (week)	48	n.a.	n.a.	5	0	0
PCL-5	–	75	6	7	0	0
VAS	–	–	30	45	0	0
BDI-II	–	47	5	8	0	1
BSI	–	3.57	0.34	0.70	0	0

The CAPS-5 interview was not administered at all time points (n.a.). Data of the PCL-5, VAS, BDI-II, and BSI questionnaires were missing at screening. VAS data were also missing at pre-treatment.

CAPS-5, Clinician-Administered PTSD scale for DSM-5; PCL-5, PTSD Checklist for DSM-5; VAS, Visual Analogue Scale; BDI-II, Beck Depression Inventory; BSI, Brief Symptom Inventory.

## 3 Discussion

The authors presented a case of a patient with severe and chronic PTSD who was treated with intensive online trauma treatment combining PE and EMDR 2.0. The results of the present case study are in line with the results from other research (18, 19) and show an intensive trauma treatment program can be applied online with success. The patient managed to complete all parts of treatment and no adverse events occurred. The PTSD symptoms showed maximum improvement and were completely absent after the intensive treatment program. During the treatment distress of all disturbing memories diminished to zero and most of the EMDR 2.0 sessions multiple target images were desensitized. Signs of dissociation were no longer present after EMDR 2.0. Mary reached full remission. She did not fulfill criteria of PTSD anymore at one month follow-up and the gains were maintained over a six month follow-up period. Moreover, she no longer suffered from severe depressive symptoms and did not report any general psychiatric symptoms anymore. The therapists did not report any issues with working online apart from a few short hiccups with the internet connection.

Strengths in this study were the use of both a clinical interview and self-report measurements for PTSD and long term follow-ups. Although the treatment was aimed at reprocessing trauma, it appeared to also have a strong effect on depressive symptoms and general psychiatric symptoms. These are remarkable results since the scoring went from severe to no symptoms at all. There are some limitations of the study: Unfortunately, measures of CPTSD were not part of the treatment measures, so, although the patient was referred to us with symptoms of CPTSD, no conclusion can be drawn about which symptoms were present, nor the severity of symptoms of CPTSD at baseline, post or follow-up. However, one can conclude that the diagnosis CPTSD was absent post treatment considering PTSD scores were 0. Moreover, earlier studies of Bongaerts (18, 19) showed that online intensive trauma treatment is also effective for patients with CPTSD. Importantly, if further research results are in line with the so far promising published data, this could have considerable advantages for psychiatric health care. For instance, it would offer more flexibility to treatment. Intensive online trauma treatment could be used in different settings (such as a forensic setting or inpatient clinics) to help patients that normally could not benefit from outpatient treatment due to safety reasons for themselves or others and it could also save travel time and costs, help overcome privacy concerns, or be made available for patients who experience physical difficulties coming to therapy.

In conclusion, this case study lends support to the idea that intensive online treatment (with PE and EMDR 2.0) can be highly effective for patients with severe and chronic PTSD and comorbid psychiatric disorders. It shows evidence for feasibility with promising results that encourage further research. Future research could include measurements of CPTSD and compare the results of online intensive treatment with regular face-to-face treatment in a bigger sample. It would be clinically relevant to be able to predict what form of therapy would tailor best to the individual needs and treatment outcome.

## 4 Patient perspective

The patient was happy with the results and reported “I’m doing very well, I’m very happy. I didn’t believe it would work so well. Sometimes I think ‘is it really true?’ I find myself just feeling very good. I really liked it online. You just see each other through the screen. It just seemed real.”

## Author’s note

Details have been altered for the purpose of anonymity. The patient provided written consent for this case study to be written.

## Data availability statement

The raw data supporting the conclusions of this article will be made available by the authors, without undue reservation.

## Ethics statement

Ethical approval was not required for the study because it was reporting on routine outcome monitoring questionnaires and no adaptations were made in that, so no formal RCT was conducted nor was the patient subject of extra questionnaires or procedures other then necessary for treatment. The study was conducted in accordance with the local legislation and institutional requirements. The participants provided her written informed consent to participate in this study. Written informed consent was obtained from the individual for the publication of any potentially identifiable images or data included in this article.

## Author contributions

SJM: Conceptualization, Data curation, Investigation, Methodology, Project administration, Supervision, Validation, Writing – original draft, Writing – review & editing. SDM: Data curation, Formal analysis, Methodology, Software, Visualization, Writing – original draft, Writing – review & editing.

## References

[1] Kessler RC Berglund P Demler O Jin R Merikangas KR Walters EE. Lifetime prevalence and age-of-onset distributions of DSM-IV disorders in the National Comorbidity Survey Replication. Arch Gen Psychiatry. (2005) 62:593–602. doi: 10.1001/archpsyc.62.6.593 15939837

[2] Steel Z Chey T Silove D Marnane C Bryant RA Van Ommeren M. Association of torture and other potentially traumatic events with mental health outcomes among populations exposed to mass conflict and displacement: a systematic review and meta-analysis. JAMA. (2009) 302:537–49. doi: 10.1001/jama.2009.1132 19654388

[3] American Psychiatric Association. Diagnostic and statistical manual of mental disorders, 5th ed. Washington, DC, USA: American Psychiatric Association Publishing (2013). doi: 10.1176/appi.books.9780890425596

[4] Cusack K Jonas DE Forneris CA Wines C Sonis J Middleton JC. Psychological treatments for adults with posttraumatic stress disorder: A systematic review and meta-analysis. Clin Psychol Rev. (2016) 43:128–41. doi: 10.1016/j.cpr.2015.10.003 26574151

[5] Lewis C Roberts NP Gibson S Bisson JI. Dropout from psychological therapies for post-traumatic stress disorder (PTSD) in adults: Systematic review and meta-analysis. Eur J Psychotraumatol. (2020) 11:1709709. doi: 10.1080/20008198.2019.1709709 32284816 PMC7144189

[6] Bongaerts H Van Minnen A de Jongh A Minnen AV Jongh D. Intensive EMDR to treat patients with complex posttraumatic stress disorder: A case series. J EMDR Pract Res. (2017) 11:84–95. doi: 10.1891/1933-3196.11.2.84

[7] Ehlers A Clark DM Hackmann A Grey N Liness S Wild J. Intensive cognitive therapy for PTSD: A feasibility study. Behav Cogn Psychother. (2010) 38:383–98. doi: 10.1017/S1352465810000214 PMC2893530 20573292

[8] Hendriks L de Kleine RA Heyvaert M Becker ES Hendriks GJ van Minnen A. Intensive prolonged exposure treatment for adolescent complex posttraumatic stress disorder: A single-trial design. J Child Psychol Psychiatry. (2017) 58:1229–38. doi: 10.1111/jcpp.12756 29057522

[9] Matthijssen SJMA Menses SDF Huisman-van Dijk HM. The effects of an intensive outpatient treatment for PTSD. Eur J Psychotraumatol. (2024) [in press]. doi: 10.1080/20008066.2024.2341548 PMC11057464 38665124

[10] Ragsdale KA Watkins LE Sherrill AM Zwiebach L Rothbaum BO. Advances in PTSD treatment delivery: Evidence base and future directions for intensive outpatient programs. Curr Treat Options Psychiatry. (2020) 7:291–300. doi: 10.1007/s40501-020-00219-7

[11] Wagenmans A Van Minnen A Sleijpen M De Jongh A. The impact of childhood sexual abuse on the outcome of intensive trauma-focused treatment for PTSD. Eur J Psychotraumatol. (2018) 9:1430962. doi: 10.1080/20008198.2018.1430962 29441153 PMC5804725

[12] Imel ZE Laska K Jakupcak M Simpson TL. Meta-analysis of dropout in treatments for posttraumatic stress disorder. J Consulting Clin Psychol. (2013) 81:394. doi: 10.1037/a0031474 PMC3893277 23339535

[13] Morland LA Wells SY Glassman LH Greene CJ Hoffman JE Rosen CS. Advances in PTSD treatment delivery: Review of findings and clinical considerations for the use of telehealth interventions for PTSD. Curr Treat Options Psychiatry. (2020) 7:221–41. doi: 10.1007/s40501-020-00215-x PMC7261035 32837831

[14] Matthijssen SJ Brouwers T van Roozendaal C Vuister T de Jongh A. The effect of EMDR versus EMDR 2.0 on emotionality and vividness of aversive memories in a non-clinical sample. Eur J Psychotraumatol. (2021) 12:1956793. doi: 10.1080/20008198.2021.1956793 34567439 PMC8462855

[15] American Psychological Association. Clinical Practice Guideline for the Treatment of Posttraumatic Stress Disorder (PTSD) in Adults (2017). American Psychiatric Association. Available online at: https://www.apa.org/ptsd-guideline/ptsd.pdf (Accessed 28 September 2022).

[16] International Society of Traumatic Stress Studies (ISTSS). New ISTSS Prevention and Treatment Guidelines (2018). Available online at: http://www.istss.org/treating-trauma/new-istssguidelines.aspx (Accessed 28 September 2022).

[17] Matthijssen SJ Brouwers TC Van den Hout MA Klugkist IG De Jongh A. A randomized controlled dismantling study of Visual Schema Displacement Therapy (VSDT) vs an abbreviated EMDR protocol vs a non-active control condition in individuals with disturbing memories. Eur J Psychotraumatol. (2021) 12:1883924. doi: 10.1080/20008198.2021.1883924 33889309 PMC8043526

[18] Alting van Geusau VVP De Jongh A Brouwers TC Moerbeek M Matthijssen SJMA. The effectiveness, efficiency, and acceptability of EMDR vs. EMDR 2.0 vs. the Flash technique in the treatment of patients with PTSD: study protocol for the ENHANCE randomized controlled trial. Front Psychiatry. (2023) 14:1278052. doi: 10.3389/fpsyt.2023.1278052 38025421 PMC10665892

[19] MOOVD. MOOVD (2020). Available online at: https://moovd.nl/.

[20] Bongaerts H Voorendonk EM van Minnen A de Jongh A. Safety and effectiveness of intensive treatment for complex PTSD delivered via home-base telehealth. Eur J Psychotraumatol. (2021) 12:1860346. doi: 10.1080/20008198.2020.1860346 34025912 PMC8128126

[21] Bongaerts H Voorendonk EM Van Minnen A Rozendaal L Telkamp BSD de Jongh A. Fully remote intensive trauma-focused treatment for PTSD and Complex PTSD. Eur J Psychotraumatol. (2022) 13:2103287. doi: 10.1080/20008066.2022.2103287 36186161 PMC9518290

[22] Boeschoten MA Bakker A Jongedijk RA Olff M. The Life Events Checklist for DSM-5 (LEC-5), Nederlandse vertaling. Diemen; Arq Academy (2014).

[23] Weathers FW Blake DD Schnurr PP Kaloupek DG Marx BP Keane TM. The Life Events Checklist for DSM-5 (LEC-5). Washington, DC: Instrument available from the National Center for PTSD (2013). Available at: www.ptsd.va.gov.

[24] Weathers FW Blake DD Schnurr P Kaloupek DG Marx BP Keane TM. Clinician administered PTSD scale – DSM 5. Washington, DC: National Centre for Posttraumatic Stress Disorder (2013).

[25] Boeschoten MA Bakker A Jongedijk RA Van Minnen A Elzinga BM Rademaker AR. Clinician administered PTSD scale for DSM-5 – Nederlandstalige versie. Diemen: Arq Psychotrauma Expert Group (2014).

[26] Boeschoten MA van der Aa N Bakker A Ter Heide FJJ Hoofwijk MC Jongedijk RA. Development and evaluation of the Dutch clinician-administered PTSD scale for DSM-5 (CAPS-5). Eur J Psychotraumatol. (2018) 9:1546085. doi: 10.1080/20008198.2018.1546085 30510643 PMC6263102

[27] Weathers FW Litz BT Keane TM Palmieri PA Marx BP Schnurr P. The PTSD Checklist for DSM-5 (PCL-5) and Life Events with extended A criterion. Washington, DC: National Center for Posttraumatic Stress Disorder (2013).

[28] Boeschoten MA Bakker A Jongedijk RA Olff M. PTSD checklist for DSM-5 and life events checklist for DSM-5 with extended A criterion – Nederlandstalige versie. Diemen: Arq Psychotrauma Expert Group (2014).

[29] Blevins CA Weathers FW Davis MT Witte TK Domino JL. The posttraumatic stress disorder checklist for DSM-5 (PCL-5): Development and initial psychometric evaluation. J Traumatic Stress. (2015) 28:489–98. doi: 10.1002/jts.22059 26606250

[30] Crichton N. Visual analogue scale (VAS). J Clin Nurs. (2001) 10:697–706.11822520

[31] Beck AT Steer RA Brown GK. Manual for the Beck Depression Inventory-II. Antonio, TX: Psychological Corporation (1996) 78:490–8. doi: 10.1037/t00742-000

[32] van der Does. BDI-II-NL. Handleiding. De Nederlandse versie van de Beck Depression Inventory-2nd edition. Lisse: Harcourt Test Publishers (2002).

[33] Derogatis LR. The Brief Symptom Inventory. Baltimore: Clinical Psychometric Research (1975).

[34] de Beurs E. Brief Symptom Inventory. Handleiding. Amsterdam: Pearson Assessment and Information (2008).

[35] Wolpe J. The Practice of Behavior Therapy. New York: Pergamon press (1990).

